# Detection of *Fusobaterium nucleatum* in feces and colorectal mucosa as a risk factor for colorectal cancer: a systematic review and meta-analysis

**DOI:** 10.1186/s13643-020-01526-z

**Published:** 2020-12-03

**Authors:** Amal Idrissi Janati, Igor Karp, Claudie Laprise, Hisham Sabri, Elham Emami

**Affiliations:** 1 Faculty of Dentistry, Université de Montréal, Montreal, Canada; 2 Department of Epidemiology and Biostatistics, Schulich School of Medicine and Dentistry, University of Western Ontario, London, Canada; 3 Department of Social and Preventive Medicine, School of Public Health, Université de Montréal, Montreal, Canada; 4 Faculty of Dentistry, McGill University, 2001 McGill College Avenue, Suite 500, Montreal, QC H3A 1G1 Canada

**Keywords:** Colorectal cancer, *Fusobacterium nucleatum*, Systematic review, Meta-analysis

## Abstract

**Background:**

Colorectal cancer (CRC) is a major cause of cancer deaths worldwide. Accumulating evidence suggests a potentially important role of colorectal infection with *Fusobacterium nucleatum* (*F*. *nucleatum*) in colorectal carcinogenesis. We conducted a systematic review, including both a qualitative synthesis and a meta-analysis, to synthesize the evidence from the epidemiological literature on the association between *F. nucleatum* detection in the colon/rectum and CRC.

**Methods:**

A systematic literature search of Ovid MEDLINE(R), Embase, Web of Science Core Collection, EBM Reviews—Cochrane Database of Systematic Reviews, and CINAHL Plus with Full Text was conducted using earliest inclusive dates up to 4 October 2020. Eligible studies were original, comparative observational studies that reported results on colorectal *F. nucleatum* detection and CRC. Two independent reviewers extracted the relevant information. Odds ratio (OR) estimates were pooled across studies using the random effects model. Newcastle-Ottawa scale was used to critically appraise study quality.

**Results:**

Twenty-four studies were included in the systematic review, of which 12 were included in the meta-analysis. Studies investigated *F. nucleatum* in feces, colorectal tissue samples, or both. In most studies included in the systematic review, the load of *F. nucleatum* was higher, on average, in specimens from CRC patients than in those from CRC-free controls. Meta-analysis showed a positive association between *F. nucleatum* detection in colorectal specimens and CRC (OR = 8.3; 95% confidence interval (95% CI) 5.2 to 13.0).

**Conclusions:**

The results of this systematic review suggest that *F. nucleatum* in the colon/rectum is associated with CRC.

**Systematic review registration:**

This systematic review protocol has been registered with the International Prospective Register of Systematic Reviews (PROSPERO) on July 10, 2018 (registration number CRD42018095866).

**Supplementary Information:**

The online version contains supplementary material available at 10.1186/s13643-020-01526-z.

## Background

Colorectal cancer (CRC) is a significant burden on global public health: it is the fourth and third most commonly diagnosed cancer in men and women [1], respectively, with more than a million new cases per year worldwide [2]. It is also the fourth leading cause of death from cancer in the world [1]. While some CRC cases are attributed to inheritance and inflammatory bowel disease, about 80% of them are sporadic [3]. Thus, identification of etiological factors is essential for efforts to reduce the morbidity and mortality from CRC.

Over the years, epidemiological studies have identified a number of CRC risk factors, such as diet, cigarette smoking, obesity, physical inactivity, diabetes, and certain genetic polymorphisms [4–11]. Furthermore, the role of some bacteria in colon carcinogenesis seems quite plausible [12, 13]. In 2011, Sears and Pardoll suggested that bacteria are the main drivers of the intestinal mucosa immune response and subsequent changes in the function and genetics of epithelial cells, which support oncogenic transformation [14]. These ideas have rapidly gained credibility due to important discoveries on the role of gut microbial dysbiosis and specifically of the bacterium *Fusobacterium nucleatum* (*F. nucleatum*) in colorectal carcinogenesis [15–25]. *F. nucleatum* is one of the dominant species of 500 or more organisms that coexist in the oral cavity [26] and the most prevalent oral species in extra-oral infections [27, 28]. Two virulence factors have been identified for *F. nucleatum*: an adhesin FadA and a self-transporting protein Fap2 [28]. On the one hand, FadA allows *F*. *nucleatum* to invade human epithelial cells, activate β-catenin signaling, induce expression of the oncogenic gene, and promote the growth of colorectal tumor cells [24, 25, 29–32]. On the other hand, the protein Fap2 inhibits the activity of immune cells and thus potentiates the progression of CCR [32, 33]. This suggests that *F. nucleatum* may participate in the colorectal tumor process and thus be a pro-oncogenic bacterium. In a murine model of CRC (APC +/−), the introduction of *F. nucleatum* increased tumor multiplicity and the selective recruitment of myeloid cells infiltrating tumors, thereby promoting tumor progression [18]. *F. nucleatum* also stimulates the recruitment of tumor-infiltrating immune cells, which generate an inflammatory microenvironment conducive to the progression of colorectal neoplasia [18]. Mouse tumors (APC +/−) exposed to *F. nucleatum* have a pro-inflammatory expression, similar to that observed in human colorectal tumors positive for *F. nucleatum* [18].

Over the last decade, many subsequent studies have reported an overabundance of *F. nucleatum* in colorectal tissues and stools from subjects diagnosed with CRC compared with CRC-free “controls.” The literature on this topic has been growing rapidly but has not yet been reviewed. We therefore conducted a systematic review and a meta-analysis to review the available literature on the association between *F. nucleatum* infection in the colon and CRC.

## Methods

This systematic review protocol has been registered with the International Prospective Register of Systematic Reviews (PROSPERO) on 10 July 2018 (registration number CRD42018095866). The protocol for this systematic review was published previously [34]. This systematic review follows the Preferred Reporting Items for Systematic Reviews and Meta-Analyses (PRISMA) guidelines as well as the Meta-analysis of Observational Studies in Epidemiology (MOOSE) guidelines (see Additional file 1 for PRISMA checklist).

### Search strategy

Literature search covered the following databases: all Ovid MEDLINE(R), Embase, Web of Science Core Collection, EBM Reviews—Cochrane Database of Systematic Reviews, and CINAHL Plus with Full Text. A comprehensive search from each database’s earliest inclusive dates (1946 for Ovid Medline, 1974 for Embase, 1945 for Web of Science, and 2008 for EBM Reviews) to 31 December 2018 was first conducted. Specific details regarding the search strategies appear in Table 1. The electronic literature search was complemented by hand-searching the list of references in the identified publications.

**Table 1:** Initial search strategy

Database and search dates	Search #1	Search #2	Search #3	Search #4	Search #5
All Ovid MEDLINE (R). January 1, 1946, to December 31, 2018	exp Colonic Polyps/ or exp Colorectal Neoplasms/	((colon$ or colorect$ or rect$ or sigmoid) adj5 (polyp$ or adeno$ or cancer$ or carcinoma$ or malignan$ or metastas$ or neoplas$ or oncolog$ or tumo$)).tw.	#1 OR #2	Fusobacterium nucleatum/ or exp Fusobacterium Infections/ or nucleatum.tw.	#3 AND #4
Embase. January 1, 1974, to December 31, 2018	exp Colon Polyp/ or exp Colorectal Tumor/	((colon$ or colorect$ or rect$ or sigmoid) adj5 (polyp$ or adeno$ or cancer$ or carcinoma$ or malignan$ or metastas$ or neoplas$ or oncolog$ or tumo$)).tw.	#1 OR #2	Fusobacterium nucleatum/ or exp Fusobacterium Infection/ or nucleatum.tw.	#3 AND #4
CINAHL Plus with Full Text. January 1, 1981, to December 31, 2018	TX ((colon* or colorect* or rect* or sigmoid) N5 (polyp* or adeno* or cancer* or carcinoma* or malignan* or metastas* or neoplas*or oncolog* or tumo*))	(MH “Colonic Polyps”) OR (MH “Colorectal Neoplasms+”)	#1 OR #2	(MH “Fusobacterium Infections+”) OR TX nucleatum	#3 AND #4
Web of Science Core Collection. January 1, 1945, to December 31, 2018	TOPIC: (((colon* or colorect* or rect* or sigmoid) NEAR/5 (polyp* or adeno* or cancer* or carcinoma* or malignan* or metastas* or neoplas* or oncolog* or tumo*)))	TOPIC: (nucleatum)	#1 AND #2		
EBM Reviews—Cochrane Database of Systematic Reviews. January 1, 2005, to December 31, 2018	((colon$ or colorect$ or rect$ or sigmoid) adj5 (polyp$ or adeno$ or cancer$ or carcinoma$ or malignan$ or metastas$ or neoplas$ or oncolog$ or tumo$)).tw.	nucleatum.tw.	#1 AND #2		

An update of the literature search was then carried out on 4 October 2020, in order to identify additional human studies that were published in French and English, since the initial search.

### Inclusion and exclusion criteria

Eligible studies were original, comparative observational studies that reported results on colorectal *F. nucleatum* infection in at least two groups: individuals diagnosed with CRC and colorectal-adenoma- and CRC-free subjects (in this article, this population will be referred to as “controls”). No demographic or geographic limitations were applied. Only studies published in English or French were included. Colorectal *F. nucleatum* had to be investigated either in feces or in biopsies from tumors in CRC patients and from healthy colorectal mucosa in “controls.” Ascertainment of *F. nucleatum* infection had to be based on microbiological detection and/or quantification tests such as any polymerase chain reaction (PCR) technique, sequencing, or microscopy visualization (e.g., fluorescence in situ hybridization technique (FISH)). Also, *Fusobacterium* had to be investigated at the species level, and the data had to be available for the particular species of *F. nucleatum*. Studies reporting data on genus or phylum levels only were thus excluded. The data on the CRC status, the outcome of interest, had to be based on laboratory-confirmed diagnosis (and thus had to be ascertained via a cancer registry or medical records).

### Study selection

Two independent reviewers (AIJ and CL) performed the study selection process based on title and abstract. Retained studies were then full-text screened by the same reviewers independently to verify the inclusion criteria. Any disagreement was resolved by discussion. If consensus could not be reached, a third reviewer determined the eligibility and approved the final list of retained studies.

### Quality assessment and data extraction

We used the Newcastle-Ottawa Scale (NOS) to assess the quality of the included observational studies. The scale includes three domains: *selection* (4 items), *comparability* (1 item), and *exposure* (3 items). A study can be awarded a maximum of one star for each numbered item within the selection and exposure categories. A maximum of two stars can be given for comparability. Study quality was then classified as poor, fair, or good, according to the Agency for Healthcare Research and Quality (AHRQ) thresholds for converting NOS scores, described as follows: (i) good quality = 3 or 4 stars in selection domain AND 1 or 2 stars in comparability domain AND 2 or 3 stars in outcome/exposure domain; (ii) fair quality = 2 stars in selection domain AND 1 or 2 stars in comparability domain AND 2 or 3 stars in outcome/exposure domain; and (iii) poor quality = 0 or 1 star in selection domain OR 0 stars in comparability domain OR 0 or 1 star in outcome/exposure domain [35–38].

The data from each study were independently extracted by two independent reviewers (AIJ and CL, AIJ and HS) and then reciprocally verified. Disagreements of two reviewers were resolved by discussion. If consensus could not be reached, a third reviewer was consulted. The following information from each article was extracted: authors’ names, country, year of publication, aim of the study, study design, study population, sample size (number of cases and number of controls), study participants’ characteristics, identification sources, criteria of matching (if any), inclusion and exclusion criteria (including any restriction of last antibiotic consumption, precursors of cancer, or inflammatory bowel disease (IBD)), localization of the tumor (colon or rectal or colorectal tumors), type of collected specimens (stools or biopsies or both), exposure definition (frequency of presence of *F. nucleatum* in specimens, relative abundance, or relative quantification of bacteria load), the technique used to detect and quantify the bacterium load, and main results and the accompanying results of statistical tests. For studies comparing more groups with controls and CRC patients (e.g., adenoma or IBD patients), only data on CRC patients and controls was extracted. Similarly, when any included study performed a second validation bacterial analysis on the same participants or on a subsample, only the results of the first technique were extracted. (See supplementary material)

### Statistical analysis

We used two approaches for data synthesis, a narrative and a quantitative synthesis using a meta-analysis. The descriptive synthesis was conducted according to the Centre for Reviews and Dissemination and included text and tables to summarize the findings.

To perform a meta-analysis, we included studies reporting any measure of association between *F*. *nucleatum* and CRC, or reporting proportions or numbers of *F. nucleatum*-positive samples in CRC cases and controls that allowed us to calculate estimates of odds ratios along with the corresponding 95% confidence intervals (CI). Then, a pooled OR estimate and its corresponding 95% CI were calculated. The data was pooled using a random effects model [39]. Heterogeneity across studies was tested using Cochran’s Q and the *I*_*2*_ statistic, and potential publication bias was investigated by visual inspection of funnel plots and Egger’s regression asymmetry test.

A subgroup meta-analysis using a random effects model was subsequently performed in order to investigate the change in *F. nucleatum* association to CRC by population area, type of colorectal specimen, and microbiological test, as well as to verify the effect of including participants with history of IBD or a recent antibiotic use in the included studies. Comprehensive Meta-Analysis Version 3 was used to conduct the meta-analysis.

## Results

### Search results

**Fig. 1:**
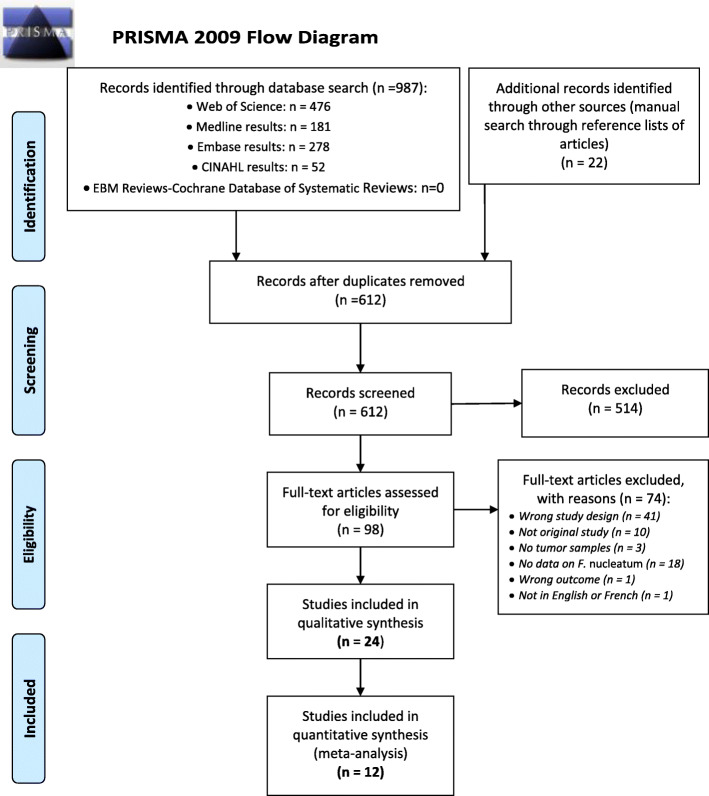
Flow diagram for selection of studies included in the systematic review and meta-analysis

Initial search (31 December 2018) and its recent update (4 October 2020) retrieved 987 records from databases and 22 additional records through manual search of relevant reviews. After removal of 397 duplicates, 612 articles were screened based on titles and abstracts, which resulted in 514 excluded articles. Ninety-eight full-text publications were assessed for eligibility. Of these, 74 were excluded for not meeting inclusion criteria. Finally, 24 studies [40–63] were included in the systematic review, of which 12 were included in the meta-analysis. Figure 1 shows the study flow diagram.

### Characteristics of studies included in the systematic review

The characteristics of the 24 studies included in the systematic review are summarized in Table 2. Studies were published between 2012 and 2020 and were mostly conducted in Asia: twelve in China, one in Japan, and two in Iran. Six studies were conducted in Europe (Germany, Spain, Italy, Ireland, Norway, and Sweden), and three in the Americas (two in USA and one in Brazil). Studies were designed as “non-nested” case-control (*n* = 21), cross sectional (*n* = 1), or nested case-control (*n* = 2), with poor or fair quality assessment of 14 studies, according to the AHRQ scale. Studies failed often in fulfilling *selection of controls* and *non-response rate* items.

**Table 2:** Characteristics of studies included in the systematic review

First author (year)	Country	Study design	No. of CRC /C	Age, mean (SD)		Exclusion of subjects with history of antibiotic use (period)	Exclusion of subjects with history of IBD diagnosis	Specimen type	Specimen collection time	Detection method	Study quality based on NOS scores
				CRC	C						
Amitay (2017) [40]	Germany	NCC	46/231	66.9 (-)	62.1 (-)	No	Yes	Feces	Before colonoscopy	16S rRNA gene analysis	Poor
Yu (2015) [41]	China	CC	42/52	---	---	Yes (last 6 months)	Yes	Feces and biopsy ^c^	Feces: non-reported Biopsies: left tissues	454 FLX pyrosequencing and PCR	Poor
Zhang (2018) [42]	China	CC	130/130	60.5 (9.8)	58.6 (8.9)	Yes (last 6 months)	Yes	Feces	Before colonoscopy	16S rDNA sequencing and PCR	Good
Flanagan (2014) [43]	Ireland	CC	7/25	---	---	No	Yes	Feces	---	qPCR	Poor
Fukugaiti (2015) [44]	Brazil	CC	7/10	65.4 (1.1)	54.8 (1.3)	Yes (unspecified)	No	Feces	Before colonoscopy	qPCR	Poor
Suehiro (2017) [45]	Japan	CC	158/60	69 (-)	32 (-)	No	No	Feces	Prior to bowel preparation	dd-PCR	Poor
Rezasoltani (2018) [46]	Iran	CC	20/31	60.9 (13.5)	59.8 (17)	Yes (last 6 months)	Yes	Feces	Before colonoscopy	qPCR	Poor
Eklof (2017) [47]	Sweden	NCC	39/65	34 (-)	34 (-)	No	Yes (for C only)	Feces	Prior to bowel preparation	qPCR	Good
Guo (2018) [48]	China	CC	215/156	---	---	Yes (last 3 months)	Yes	Feces	Time of diagnosis, before resection	qPCR	Poor
Liang (2017) [49]											
Cohort I	China	CC	170/200	67.2 (11.6)	59.3 (5.8)	Yes (last 3 months)	No	Feces	Before or one month after colonoscopy	qPCR	Good
Cohort II	China		33/36	63.4 (9.6)	53.2 (12.2)						
Mira-Pascual (2015) [50]	Spain	CC	7/9	71.1 (10.1)	52.6 (15.2)	No	No	Feces and biopsy^d^	≥ 1 week before colonoscopy	qPCR	Good
Repass (2018) [51]	USA	CC	40/40	---	---	No	No	Biopsy	During surgery	qPCR	Fair
Russo (2017) [52]	Italy	CC	10/10	---	---	Yes (last 3 months)	No	Feces	1 day before surgery for CRC and unspecified for C	qPCR	Poor
Vogtmann (2016) [53]	USA	CC	52/52	61.8 (-)	61.2 (-)	No	No	Feces	Prior to surgery or other treatment	WGSS	Good
Wang (2016) [54]	China	CC	10/10	54.8 (-)	54 (-)	No	No	Feces	---	PCR	Good
Wang (2012) [55]	China	CC	46/56	60^a^	49^a^	Yes (last 3 months)	Yes (for C only)	Feces	Before surgery for CRC patients and unspecified for C	qPCR	Poor
Wong (2017) [56]	China	CC	104/102	66.9 (10.1)	57.1 (5.8)	Yes (last 3 months)	Yes	Feces	Prior to bowel preparation for colonoscopy	qPCR	Poor
Wu (2013) [57]	China	CC	19/20	58.3 (8.7)	53.2 (5.4)	Yes (last 3 months)	Yes (for C only)	Feces	---	qPCR	Good
Xie (2017) [58]	China	CC	327/242	63.5 (10.2)	60.1 (8.4)	Yes (last month)	No^b^	Feces	Prior to bowel preparation for surgery or endoscopy	qPCR	Good
Yu (2016) [59]	China	CC	93/20	59.25(-)	---	No	No	Biopsy	Colonoscopy or surgery	FISH	Poor
Yu (2017) [60]	China	CC	74/54	63 (50.7)	67 (34.9)	Yes (last 3 months)	Yes	Feces	Before or mostly after colonoscopy	Metagenomic sequencing	Poor
Tunsjo (2019) [61]	Norway	CC	23/22	70 (-)	57 (-)	No	No	Feces and biopsy ^e^	Prior to bowel preparation or 1 week after colonoscopy	qPCR	Poor
Liu (2020) [62]	China	CC	53/45	52.4 (18.8)	53.7 (16.7)	Yes (last month)	Yes	Feces	Prior to bowel preparation for colonoscopy	qPCR	Good
Kashani (2020) [63]	Iran	CS	35/45	---	---	No	No	Biopsy	Colonoscopy	PCR	Satisfactory^f^

*No.* number, *CRC* colorectal cancer cases, *C* controls (colorectal-adenoma- and CRC-free subjects), *IBD* inflammatory bowel disease, *NCC* nested case-control, *CC* case-control, *CS* cross sectional, *qPCR* quantitative polymerase chain reaction, *NOS* Newcastle-Ottawa scale, *WGSS* whole genome shotgun sequencing, *dd-PCR* droplet digital PCR, *FISH* fluorescent in situ hybridization

--- Not reported

^a^Median

^b^Controls were those with normal or chronic inflamed colorectal mucosa

^c^Biopsies were taken from only 31/42 CRC and 37/52 C

^d^Biopsies were taken from 7/7 CRC and 5/9 C

^e^Biopsies were taken from 21/23 CRC and 11/22 C

^f^In NOS adapted for CS studies, the study quality is based on the total score and rated as follow: very good (9–10 points), good (7–8 points), satisfactory (5–6 points), and unsatisfactory (0 to 4 points)

In seven studies, cases and controls were matched for two to four variables, including age, gender, body mass index, ethnicity, and the time period of sample collection [42, 47, 50, 51, 53, 54, 57, 62]. There were 13 studies that excluded subjects with reported antibiotic use in the last month, or in the last 3 months or 6 months, and 11 studies that excluded patients previously diagnosed with IBD.

The majority of studies investigated *F. nucleatum* in feces only (18 studies), while three studies analyzed biopsies only, and another three studies analyzed both types of specimens. Quantitative PCR was the most used bacteria-detection technique followed by sequencing techniques, while only one study used FISH technique.

In most of the included studies, feces were collected before colonoscopy or surgery, except for the study by Yu et al. [60] where feces were collected more after colonoscopy than before. Tunsjo et al. [61] also reported collecting feces either before colonoscopy or 1 week after. In four studies, no information was provided about the timing of specimen collection [43, 54, 57].

### Comparison of *Fusobacterium nucleatum* load in colorectal specimens between colorectal cancer cases and controls

As shown in Table 3, *F. nucleatum* quantification (load) in colorectal specimens was reported by 18 studies [41–50, 52, 53, 55, 56, 58, 60–62] including one study with two independent cohorts [49]. Bacteria quantification was mostly performed in stool specimens, except for Yu et al. [41] who quantified the bacteria in both feces and biopsies. Vogtmann et al. [53], Wang et al. [55], and Zhang et al. [42] reported the relative abundance (RA) of the bacteria as a percentage, corresponding to the contribution of *F. nucleatum* to the total bacteria present in specimens [42, 53, 55]. Their results confirmed that *F. nucleatum* does not naturally contribute to a healthy gut microbiome (RA varied from 0.001 to 0.003% in controls). When investigated in CRC case specimens, *F. nucleatum* was significantly more abundant than in controls, but still in very small proportions (RA varied from 0.061 to 0.17%). While only four studies reported results of absolute quantification of *F. nucleatum*, either as copy number or bacteria counts, the majority of studies performed relative quantification (RQ) of *F. nucleatum* to the total bacteria present in specimens based on the ΔΔCq method. These RQ studies reported a significantly higher *F. nucleatum* load in colorectal specimens of CRC patients compared to controls, except for two studies [50, 52]. Fold change in *F. nucleatum* from controls to CRC cases was estimated by three studies with very different values: 132-fold according to Wong et al. [56], 66-fold according to Tunsjo et al. [61], and 5.2-fold reported by Xie et al. [58].

**Table 3:** Studies comparing *F. nucleatum* load in colorectal specimens of colorectal cancer cases and controls

First author (year)	Specimen type (No. of specimens CRC/C)	***F. nucleatum*** quantification measures reported	Statistics reported	***F. nucleatum*** load in CRC compared to C		
				C	CRC	*p*
**Relative abundance**						
Zhang (2018) [42]	Feces (130/130)	Relative contribution to total bacteria (%)	Mean	0.001	0.17	< 0.001
Vogtmann (2016) [53]	Feces (52/53)	Relative contribution to total bacteria (%)	Mean	0.003	0.08	0.043
Wang (2012) [55]	Feces (46/56)	Relative contribution to total bacteria (%)	Mean	0.002	0.061	0.005
**Absolute quantification**						
Yu (2017) [60]	Feces (74/54)	Bacteria counts	Rank mean	45.09 IMG 40.32 MLG 54.62 OTU	78.66 IMG 82.14 MLG 71.71 OTU	< 0.001
Suehiro (2017) [45]	Feces (158/60)	Absolute copy number of *F. nucleatum*	Median (min–max)	17.5 (0–5793)	317 (0–17,343)	< 0.0001
Fukugaiti (2015) [44]	Feces (7/10)	Log no. copies of *F. nucleatum*/gram	Mean ± SD (min-max)	4.0 ± 1.5 (1.0–6.4)	6.2 ± 1.5 (3.5–8.0)	0.01
Mira-Pascual (2015) [50]	Feces (6/10)	Log no. gene copies of *F*. *nucleatum*/mg	Median (IQR)	4.16 (3.47–4.85)	4.70 (3.85–5.15)	---
Rezasoltani (2018) [46]	Feces (20/31)	C_q_ value (Quantity of *F. nucleatum* = 10 ($\frac{Cq-b}{m}$ ))	Mean (SD)	29.16 (3.31)	17.74 (3.59)	< 0.05
Liu (2020) [62]	Feces (53/45)	Log 10 copies of *F. nucleatum* ∕ gram	Mean (SD)	≈3.5 (1)	≈6 (1)	< 0.01
**Relative quantification**						
Russo (2017) [52]	Feces (10/10)	Ratio: $\frac{Cq\kern0.5em F. nucleatum\kern0.75em }{Cq\ total\ bacteria}$	Mean	≈2.25	≈1.8	> 0.05
Yu (2015) [41]	Feces (42/52)	ΔC_q_ value	Median [IQR]	≈26 [2]	≈19 [2]	< 0.001
	Biopsy (31/37)			≈27 [2]	≈18 [3]	< 0.001
Flanagan (2014) [43]	Feces (7 /25)	2^−ΔCq^ value	Median	2^−21^	2^−15^	0.02
Liang (2017) [49] Coh I	Feces (170/200)	2^−ΔCq^ value	Median	≈2^−17^	≈2^−5^	< 0.0001
Liang (2017) [49] Coh II	Feces (33/36)	2^−ΔCq^ value	Median	≈2^−16^	≈2^−13^	0.012
Eklof (2017) [47]	Feces (39/66)	Log 2^−ΔCq^	Median [IQR]	≈− 10 [2]	≈− 7 [5]	< 0.001
Guo (2018) [48]	Feces (215/156)	Log 2^−ΔCq^	Mean	≈−4	≈− 3	< 0.0001
Wong (2017) [56]	Feces (104/102)	Log 2^−ΔCq^ (**fold change from HC to CRC**)	Mean	≈−7	≈−5 **(132)**	< 0.001
Xie (2017) [58]	Feces (327/242)	Log_2_ RQ, based on ΔCq (**Fold change from HC to CRC**)	Mean	≈−20	≈− 16 for early stage and −18 for advanced stage **(5.12)**	0.006 < 0.001
Tunsjo (2019) [61]	Feces (23/22)	2^−ΔCq^ value (**fold change from HC to CRC**)	Median	≈2^−7^	≈2^−5^ **(66)**	0.0073

*CRC* colorectal cancer cases, *C* controls (colorectal-adenoma- and CRC-free CRC and C subjects), *SD* standard deviation, *Coh* cohort, *OTUs* operational taxonomic units, *MLGs* metagenomic linkage groups, *IMG* integrated microbial genome, *C*_*q*_ quantification cycle in qPCR, *ΔC*_*q*_ the average Cq value of *F. nucleatum*—the average Cq value of total bacteria (or reference gene), *min* minimum, *max* maximum

---Not reported

**≈**A value read from a graph in the study material

### Comparison of frequency of presence of *Fusobacterium nucleatum* in colorectal specimens between colorectal cancer cases and controls

Rather than absolute or relative quantification of *F. nucleatum* load, some studies compared the frequency of presence of the bacteria in colorectal specimens between controls and CRC patients, as shown in Table 4. Some studies [40, 44, 49–51, 53, 54, 57, 60, 61, 63] reported the frequency of presence of the bacterium in colorectal specimens when the bacterium was simply detected (by PCR, sequencing, or FISH techniques), while other studies [45, 47, 58] reported the frequency of presence of the bacterium when its load level was above a specific cutoff value. The cutoff values were typically set to the values that served to achieve the highest discrimination between CRC patients and controls in terms of Youden index. Tunsjo et al. [61] set a cutoff value for detecting *F. nucleatum* in feces, but not in biopsies. As shown in Table 4, the cutoff value was not reported in one study [58] and varied between the three others: 260 copies of *F. nucleatum* by Suehiro et al. [45] and a 2^−ΔCq^ of 0.00026 (2^−12^) for both Eklof et al. [47] and Tunsjo et al. [61].

**Table 4:** Frequency of presence of *F. nucleatum* in CRC and controls’ specimens and odds ratio for the association between *F. nucleatum* and colorectal cancer

First author (year)	Specimen type (no. of specimens CRC/C)	Definition of specimen positive to ***F.*** nucleatum	Prevalence of ***F. nucleatum*** (+)			OR [95% CI]	Adjusted OR [95% CI]
			C (%)	CRC (%)	***p***		
Amitay (2017) [40]	Feces (46/231)	*F. nucleatum* is detected	20	51	< 0.001	4.16 [2.15–8.07]	---
Fukugaiti (2015) [44]	Feces (7/10)	*F*. *nucleatum* is detected	90	100	---	2.37 [0.08–66.88]	---
Liang (2017) [49] Coh I	Feces (170/200)	*F. nucleatum* is detected	72	98.2	< 0.0001	21.22 [6.57–68.5]	---
Mira-Pascual (2015) [50]	Feces (7/9)	*F. nucleatum* is detected	22.2	85	---	19.86 [1.47–268.17]	---
	Biopsy (7/5)	*F. nucleatum* is detected	0	26,6	---	4.6 [0.18–121.18]	---
Vogtmann (2016) [53]	Feces (52/52)	*F. nucleatum* is detected	26.9	63.5	0.0002	4.72 [2.05–10.85]	---
Wang (2016) [54]	Feces (10/10)	*F. nucleatum* is detected	10	60	---	13.5 [1.2–152.21]	---
Wu (2013) [57]	Feces (19/20)	*F. nucleatum* is detected	0	68.4	---	85.15 [4.42–1638.85]	---
Yu (2017) [60]	Feces (74/54)	*F*. *nucleatum* is detected	3.7	52.7	7.53E-08	29 [6.57–128]	---
Kashani (2020) [63]	Biopsy (35/45)	*F*. *nucleatum* is detected	24	68	0.0001	6.74 [2.5–18.07]	---
Repass (2018) [51]	Biopsy (40/40)	The product is amplified in the qPCR reaction	5	40	---	12.67 [2.67–60.05]	---
Yu (2016) [59]	Biopsy (93/20)	Average number of bacteria per field ≥ 5, visualized by FISH technique	Invasive 20, in biofilms 10	Invasive 65.9^a^, in biofilms 48.4^a^	< 0.05	7.73 [2.38–25.07] ^e^	---
Tunsjo (2019) [61]	Feces (23/22)	*F. nucleatum* detected with ΔCq values < 12	0	35	---	24.9 [1.33–463.72]	---
	Biopsy (21/11)	*F*. *nucleatum* is detected	18	52	---	4.93 [0.85–28.67]	---
Suehiro (2017) [45]	Feces (158/60)	*F. nucleatum* detected at a higher level based on cutoff value (> 260 copies )^b^	10	54	---	10.57 [4.3–25.98]	---
Eklof (2017) [47]	Feces (39/65)	*F. nucleatum* detected at a higher level based on cutoff value (2^−ΔCq^ > 0.00026^c^≈**2**^**−12**^ )^c^	24.3	69.2	---	7 [2.89–16.96]	---
Xie (2017) [58]	Feces (327/242)	*F. nucleatum* detected at a higher level based on cutoff value (value is not reported) ^d^	---	---	---	4.31 [2.96–6.28]	4.28 ^f^ [2.27–8.09]

*no.* number, *CRC* colorectal cancer cases, *C* controls (colorectal-adenoma- and CRC-free subjects), *OR* odds ratio

---Not reported

^a^The value was calculated as the average of reported prevalence in proximal and distal separately

^b^260 copies of *F. nucleatum* was the best cutoff point to discriminate between HC and CRC in receiver operating characteristic (ROC) analysis resulting in estimated sensitivity of 54% and estimated specificity of 90%, and the area under the ROC curve was 0.75

^c^The cutoff value of 0.00026 (**= 2**^**−ΔCq**^ **= 2**^**−12**^) gave the most reliable analysis for detecting cancer in the study patients, with estimated specificity of 76.9%

^d^ROC curve was used to evaluate the diagnostic value of bacterial candidates in distinguishing CRC and controls and to determine the best cutoff values that maximized the Youden index

^e^OR was calculated based on prevalence of invasive *F*. *nucleatum* (in tissues) as its detection was higher than within biofilms

^f^OR adjusted for age, gender, FIT test, two additional bacteria markers, history of diabetes, and high blood pressure

*F. nucleatum* was commonly detected in all specimens across studies, except for Mira-Pascual et al. and Wu et al., who did not detect the bacterium in controls’ biopsies and controls’ feces respectively [50, 57]. The frequency of specimens positive to *F. nucleatum* was higher among CRC patients than controls in all the studies. The study by Yu et al. was the only one to use FISH technique, which allowed for quantifying the bacteria within tissues (called invasive *F. nucleatum*) and in the biofilm separately. Their results showed a higher frequency of presence of *F. nucleatum* in tissues than in biofilm.

### Association between *Fusobacterium nucleatum* and colorectal cancer

**Fig. 2:**
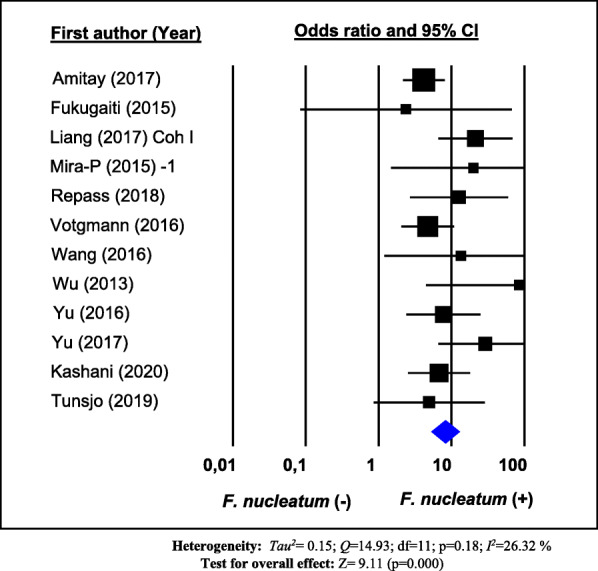
Forest plot of the association between *F. nucleatum* in colorectal specimens and colorectal cancer. Results of a random-effects meta-analysis of 12 observational studies

**Fig. 3:**
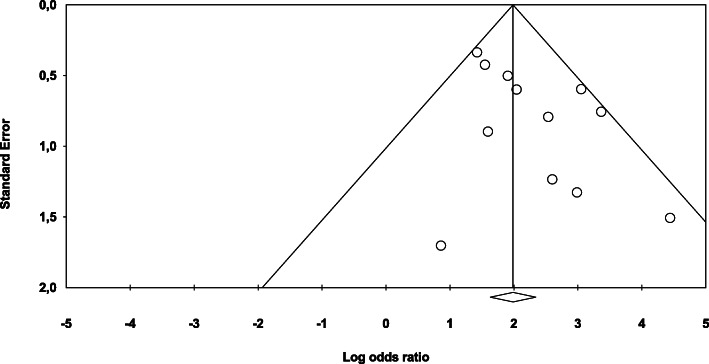
Funnel plot of the natural logarithm-transformed odds ratio estimates, by the corresponding standard error. Circles, studies in the meta-analysis

To perform a meta-analysis on the association between *F. nucleatum* and CRC, we pooled data from 12 studies [40, 44, 49–51, 53, 54, 57, 59–61, 63] that operationally defined the presence of *F. nucleatum* in terms of the detection of the bacterium in colorectal specimens, with no use of a cutoff value that optimizes distinction between cases and controls, as described above. As shown in Fig. 2, the overall pooled OR and the corresponding 95% CI estimated in a random effects model show a positive association between *F. nucleatum* detection in colorectal specimens and CRC (OR = 8.3; 95% confidence interval (95% CI) 5.2 to 13.0), with moderate heterogeneity (*I*_*2*_ = 26.32%, *p* value for heterogeneity = 0.18). Funnel plot for investigating publication bias is presented in Fig. 3. Visual inspection of the funnel plot does not suggest an evident publication bias, which was also confirmed by Egger’s regression test (*p = 0.053*). Adjusted pooled OR estimate could not be calculated due to non-availability of adjusted OR estimates from the reports of the individual studies.

**Fig. 4:**
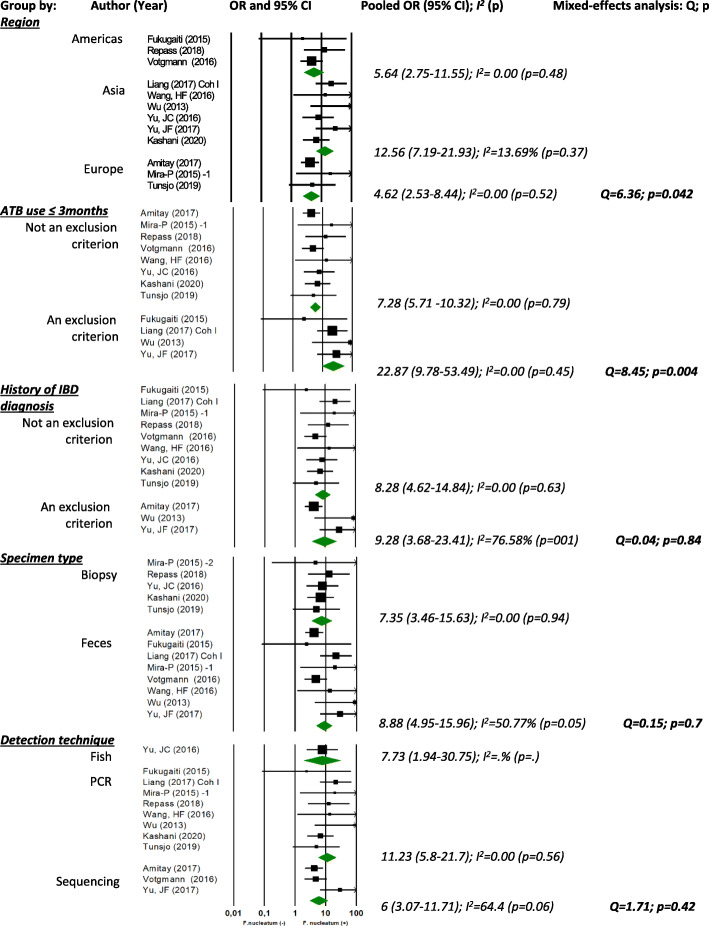
Forest plots for subgroup meta-analysis. ATB, antibiotic; IBD, inflammatory bowel disease; OR, odds ratio

Subgroup meta-analysis shows a stronger association between *F. nucleatum* and CRC in Asiatic populations, compared to European and American populations, as well as in studies excluding subjects with reported antibiotic use in the last 3 months, compared with studies that did not exclude these subjects (Fig. 4). However, the association was not statistically significantly different by specimen type (stools vs. biopsies), bacterial detection technique (FISH vs. qPCR vs. sequencing), or previous IBD diagnosis as exclusion criteria for study participation.

## Discussion

This systematic review summarizes results from 24 observational studies that compared the prevalence of the presence of *F. nucleatum* and/or the mean/median of *F*. *nucleatum* load in colorectal specimens, among cases of CRC and controls. Studies used mainly two ways to compare CRC patients and controls in regard to colorectal infection by *F. nucleatum*: (1) bacterium load expressed by RA (a percentage expressing the relative contribution of *F. nucleatum* to total bacteria), absolute quantification (bacteria count), or more often by RQ (2^−ΔCq^ value by qPCR technique, with ΔC_q_ = (the average Cq value of *F. nucleatum* − the average Cq value of total bacteria or reference gene)); and (2) frequency of the presence of *F. nucleatum* in colorectal specimens.

It is true that RA and RQ are both relative measures of the bacterium load, but many studies used one or the other term to express the same thing, which can be confusing. Thus, in this systematic review, we tried to differentiate between the two terms, RA and RQ, and represent results accordingly. This showed that RA was used less commonly than RQ, even if RA also allows better comparison between healthy and altered microbiome composition, since dysbiosis is, by definition, the loss in representation of different bacterial phyla within the whole bacterial composition of the microbiome.

We also mention some issues with publishing data of *F. nucleatum* RQ in individual studies. Most of the time, RQ was extracted from papers’ supplementary tables or graphs that were often poorly annotated. Also, even if individual studies based their RQ on a common ΔΔCq method, values were reported differently across studies. Thus, we encourage researchers to standardize the way to report RQ data.

Overall, results of absolute and relative quantification of *F. nucleatum* were higher in CRC cases compared to controls across most studies. Only three studies reported the fold change of *F. nucleatum* load from controls to CRC cases, but one was much larger than the others: 5.12-fold by Xie et al. [58], 66-fold by Tunsjo et al. [61], and 132-fold by Wong et al. [56]. When comparing these studies, Xie et al. [58] included subjects with chronic inflamed colorectal mucosa within their control group, while Wong et al. [56] excluded subjects with IBD; Tunsjo et al. [61], for their part, did not report excluding IBD patients from participation. Presence of IBD patients among controls in the study of Xie et al. [58] could probably have blurred the difference in *F*. *nucleatum* load between their CRC and controls. In this regard, we mention a study by Strauss et al. who isolated *Fusobacterium* spp. from 63.6% of patients with gastrointestinal disease compared to 26.5% of healthy controls (*P* = 0.01), with *F. nucleatum* representing 69% of recovered *Fusobacterium* spp. in their IBD patients [64].

Our meta-analysis included 12 studies based on a total of 1098 cases and 1069 controls. Only crude pooled OR could be calculated, and it shows an association between the presence of F. *nucleatum* in feces or colorectal mucosa and CRC.

All included studies reported results of *F. nucleatum* detection in specimens collected just before colonoscopy or surgery. Furthermore, the estimated OR for the association between *F. nucleatum* and CRC was not adjusted for potential confounders. Thus, causal explanation of the “positive” empirical association (as quantified by the pooled-OR estimate) is not warranted, in our view. However, the involvement of *F. nucleatum* in early CRC carcinogenesis stages has been suggested by other studies that identified the bacterium in precancerous lesions. Its RA was reported to be higher in adenomas than in healthy tissues and lower in adenomas than in carcinomas, reflecting a gradual enrichment of the colon with *F. nucleatum* in parallel to the adenoma-carcinoma sequence [65–67]. The level of *F. nucleatum* also seems to increase with advancing stages of dysplasia [43].

Subgroup meta-analysis suggested (even if weakly) that the *F. nucleatum*–CRC association (if it does exist) may be stronger in Asian populations than in American or European ones. This finding seems to be in line with the results of a recent meta-analysis by Huang et al. [68] on the diagnostic value of fecal *F. nucleatum* in screening CRC, which had a better performance in Asians. The apparent dependence of the association between *F. nucleatum* and CRC on population area may be explained by lifestyle differences between populations and/or by diversity in human gut microbiomes at the population level. Nishijima et al. analyzed gut microbiomes of Japanese individuals by comparing metagenomic data obtained from 106 Japanese subjects with those from 11 other nations. They found that gut microbiome of the Japanese is considerably different from those of other populations and cannot be explained by diet alone [69].

We also found that the estimated association between *F. nucleatum* and CRC was stronger in the subgroup of studies that excluded subjects with recent antibiotic use, compared with the subgroup of studies that did not. This can be explained by a possible bias due to introducing subjects with microbiomes altered by recent antibiotic use.

Some studies failed in reporting critical information, such as time of specimen collection, which was not reported by four studies [41, 43, 54, 57]. Also, in one study [60], feces were collected most frequently after colonoscopy. However, colonic microbiota has been shown to be disturbed by the bowel cleansing protocol and takes about 2 weeks to recover to its original composition depending on the cleansing protocol [70].

*F. nucleatum* is a very heterogeneous species of the Fusobacteria phylum and has been classified into four to five subspecies: *animalis*, *nucleatum*, *polymorphum*, *vincentii/fusiforme*. *F. nucleatum*, subsp. *nucleatum*, is mainly isolated in periodontal pathological sites, while *F. nucleatum* subsp. *vincentii/fusiforme* is often isolated from healthy sites as normal flora. *F. nucleatum* subsp. *animalis* and *polymorphum* are associated with complications of pregnancy, and *F. nucleatum* subsp. *animalis* is associated with inflammatory bowel disease [71]. xIn our systematic review, data about subspecies of F. *nucleatum* was only reported by the study of Amitay et al. [40], in which the four subspecies were identified: ssp. *nucleatum*, *animalis*, *vincentii*, and *polymorphum*. In a study by Ye et al., five *F. nucleatum* subspecies were identified in clinical CRC specimens, with ssp. *animalis* being the most common one [72]. Komiya et al. examined whether identical strains of *F. nucleatum* could be isolated from colorectal and saliva specimens from the same patient. Saliva and colorectal specimens were analyzed from 14 CRC patients by qPCR, of which 40% exhibited identical strains of *F. nucleatum* in their colorectal and saliva specimens [73].

The oral cavity can serve as a reservoir for the systemic dissemination of pathogenic bacteria and their toxins, leading to infections and inflammations in distant bodily sites. Several oral species were identified in infections at extraoral sites. Han et al. [28] suggested a spread of oral infection due to transient bacteremia leading to bacterial colonization in extraoral sites, systemic damage by toxins free of oral pathogens, and systemic inflammation caused by soluble antigens of oral pathogens. *F. nucleatum* is one of the most dominant species of the oral microbiota [26]. It often aggregates with other oral bacteria and plays an essential role in the formation of dental plaque, acting as a bridge between early colonizing bacteria (Gram-positive bacteria) and late colonizing bacteria (Gram-negative bacteria) [74]. Such a mechanism resembles the proposed *driver-passenger model* in explaining how bacteria in the intestinal microbiota could be involved in carcinogenesis. The first step consists of colonization of the intestine by pathogenic bacteria known as “drivers” with pro-inflammatory and pro-carcinogenic potential (B. *fragilis* and E. *coli* in particular). The tumor progression would then cause a modification in the tumor microenvironment, allowing colonization by opportunistic bacteria known as “passengers” (*F. nucleatum* and *Streptococcus gallolyticus* in particular), promoting further development of the tumor [75, 76]. The bacterial “drivers” and “passengers” would thus have distinct temporal roles in the pathogenesis of CRC [75]. This model implies that there is not a single bacterium that would alone be incriminated in the occurrence and development of CRC, but rather a bacterial community whose taxonomic composition continues to change throughout the tumorigenic process, thereby allowing specific bacteria to play their role in tumor transformation, according to their virulence and other properties. Moreover, some believe that the oral bacterium *F. nucleatum* plays a role in the development of CRC within a bacterial community or biofilm, rather than as an individual pathogen [77]. Warren et al. analyzed the bacterial composition of 130 colorectal tumors and their surrounding healthy tissues, and confirmed the over-representation of *Fusobacterium*, but in the simultaneous presence of two other commensal oral bacteria, *Leptotrichia* and *Campylobacter*, in individual tumors [22].

## Conclusion

The results of this systematic review and meta-analysis suggest that the *F. nucleatum* in feces or colorectal mucosa is associated with CRC. Future clinical and epidemiological studies should address the potential role of *F. nucleatum* in the etiology of CRC. Further, the bacterium should be investigated in the colon at the subspecies level to assess the oral origin of colorectal infection with *F. nucleatum.*

## Supplementary Information

**Additional file 1.** PRISMA 2009 checklist.

**Additional file 2.** Supplementary material.

## Abbreviations

CRC: Colorectal cancer
*F. nucleatum*: *Fusobacterium nucleatum*
95% CI: 95% confidence interval
OR: Odds ratio
PRISMA: Preferred Reporting Items for Systematic Reviews and Meta-analyses
MOOSE: Meta-analysis of Observational Studies in Epidemiology
NOS: Newcastle-Ottawa scale
AHRQ: Agency for Healthcare Research and Quality
RA: Relative abundance
RQ: Relative quantification
IBD: Inflammatory bowel disease
PCR: Polymerase chain reaction
qPCR: Quantitative polymerase chain reaction
FISH: Fluorescence in situ hybridization
